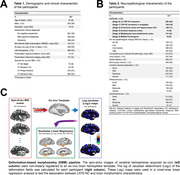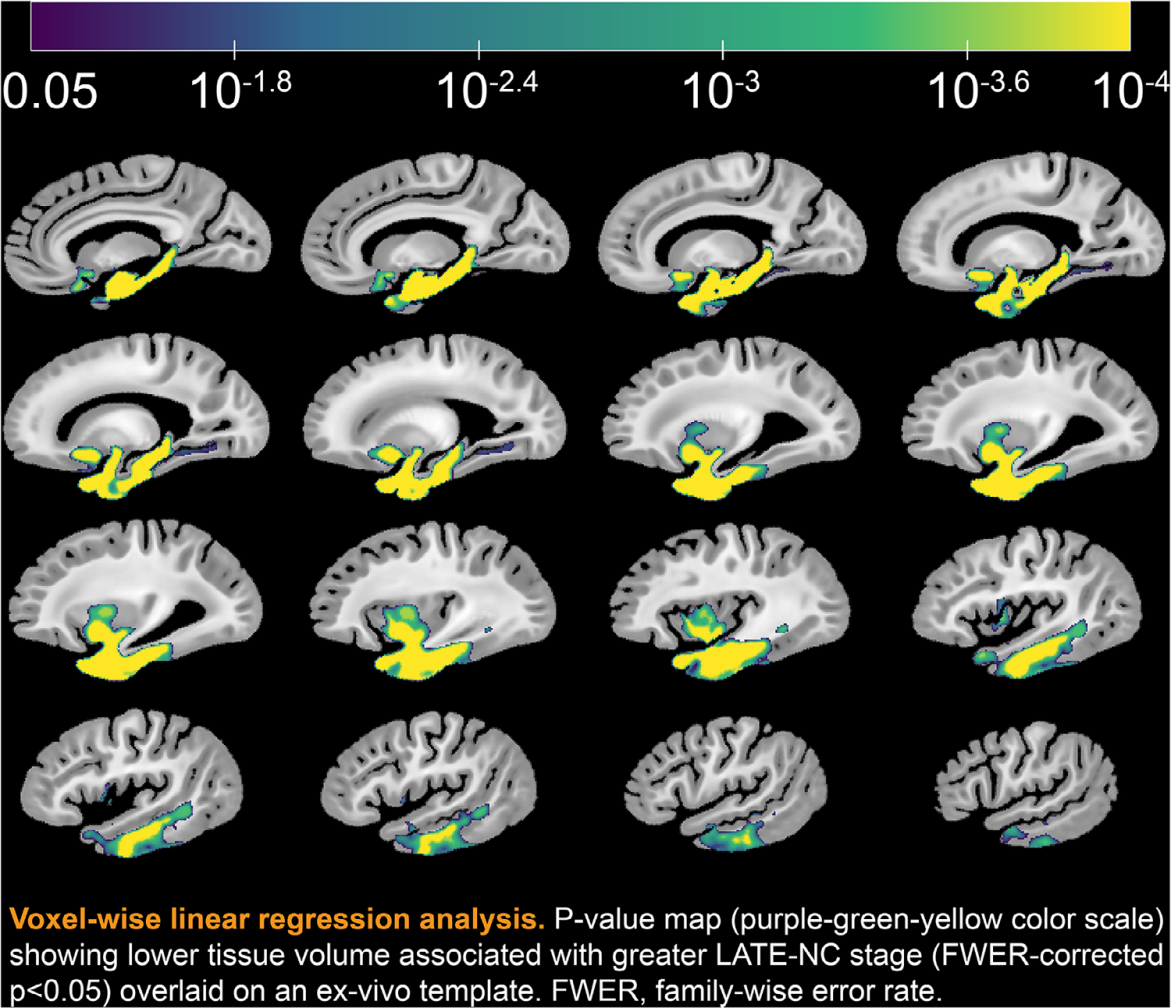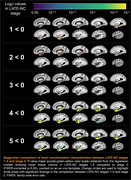# Limbic predominant age‐related TDP‐43 encephalopathy neuropathological change (LATE‐NC) is associated with lower volume in gray and white matter of the temporal and frontal lobes and basal ganglia: A deformation‐based brain morphometry study

**DOI:** 10.1002/alz.083671

**Published:** 2025-01-09

**Authors:** Mahir Tazwar, Arnold M Evia, Abdur Raquib Ridwan, David A. Bennett, Julie A. Schneider, Konstantinos Arfanakis

**Affiliations:** ^1^ Illinois Institute of Technology, Chicago, IL USA; ^2^ Rush University Medical Center, Chicago, IL USA

## Abstract

**Background:**

Limbic‐predominant age‐related TDP‐43 encephalopathy neuropathological change (LATE‐NC) is common in older adults and has been associated with substantial cognitive impairment. However, the association of LATE‐NC with brain morphometry has not been thoroughly investigated. In this work, we examined the association of LATE‐NC with brain morphometric anomalies using deformation‐based morphometry (DBM) in a large community cohort of older adults that came to autopsy (N=897).

**Method:**

Cerebral hemispheres were acquired from 897 deceased older adults participating in Rush Memory and Aging Project, Religious Orders Study, Minority Aging Research Study, and Clinical Core. Hemispheres were imaged ex‐vivo on 3T MRI scanners, which was followed by detailed neuropathologic examination. The participant scans were non‐linearly registered to an ex‐vivo template, and the resulting deformation fields were used to calculate the logarithm of the Jacobian determinant in each voxel (LogJ maps).

Voxel‐wise linear regression was used to test the association between deformations shown in the LogJ maps and LATE‐NC stages, controlling for other age‐related neuropathologies (Alzheimer’s disease, Lewy bodies, arteriolosclerosis, atherosclerosis, cerebral amyloid angiopathy, gross and microscopic infarcts), age at death, sex, education, postmortem intervals, and scanners. To identify the earliest LATE‐NC stage exhibiting morphometric abnormalities, LogJ values were compared between LATE‐NC stages 1–5 and stage 0. Statistical significance was set at p<0.05.

**Result:**

Voxel‐wise analysis revealed an independent association of LATE‐NC with significantly lower volume in both gray and white matter regions within the temporal and frontal lobes and basal ganglia (p<0.05), including amygdala, hippocampus, entorhinal, parahippocampal, temporal pole, inferior temporal, middle temporal, fusiform, medial orbitofrontal, lateral orbitofrontal, insula, accumbens, and putamen cortices. Groupwise comparison of LogJ values revealed significant morphometric anomalies in small temporal lobe areas in stages 1–2, more temporal lobe as well as basal ganglia tissue in stage 3, and finally also included frontal lobe areas in stages 4–5.

**Conclusion:**

The morphometric anomalies were detected as early as LATE‐NC stage 1, suggesting that MRI is sensitive to the early stages of the disease. This pattern is consistent with the known pathological distribution of LATE‐NC in the brain and may potentially be used in the development of a marker of this devastating neuropathology.